# A Primary Care Group Resilience Intervention Promotes Child and Caregiver Behavioral Health

**DOI:** 10.2196/67638

**Published:** 2025-02-10

**Authors:** Joan Jeung, Andrew Nguyen, Jennifer Martinez, Li Zhang

**Affiliations:** 1 Department of Pediatrics, Division of Developmental Medicine, University of California San Francisco (UCSF) School of Medicine, 675 - 18th St, 2nd Floor, UCSF Box 3132, San Francisco, CA, 94143, United States, 1 (415) 353-2080, 1 (415) 502-0014; 2 Department of Medicine, University of California San Francisco (UCSF) School of Medicine, San Francisco, CA, United States; 3 Graduate Medical Sciences, Chobanian & Avedisian School of Medicine, Boston University, Boston, MA, United States

**Keywords:** parenting education, parent-child relationship, adverse childhood experiences, child behavior, children, caregiver, caretaker, parenting, family, stress, anxiety, behavior, relational health, psychoeducation, psychological education, resilience intervention, group-based, pilot study

## Abstract

This pilot study of the redesigned Resilience Clinic, a group-based psychoeducational intervention designed to promote relational health and child and family resilience provides preliminary evidence that participation in this intervention is associated with decreased caregiver stress, anxiety, and child behavioral concerns.

## Introduction

Since adverse childhood experiences (ACEs) including child maltreatment, family violence, parental substance abuse, and parental mental illness may increase health and behavioral risks [1,2], further research into preventive early childhood interventions, before the onset of ACE-associated sequelae is required [3]. The Resilience Clinic (RC) [4] is a primary care-based group psychoeducational intervention promoting resilience among children exposed to significant adversity. Initially serving children of all ages, this program was redesigned based on significant parent feedback to focus on early childhood (ages 0‐5); the curriculum incorporates Circle of Security-Parenting (building secure attachment) [5] and Dovetail Learning (mindful stress management) [6] in 6 weekly, hour-long group sessions. This study explored whether participation in RC decreased measures of (1) caregiver stress, anxiety, and depression, and (2) child behavioral challenges.

## Methods

This pilot study analyzed pre-post differences in caregiver-reported measures of behavioral health after RC participation. Eligibility criteria included children aged 0‐5, referred by primary care providers following positive ACE screening; siblings were excluded. Eligible participants were allowed to join the intervention without joining the study.

Study measures included the Child Behavior Checklist (CBCL) for ages 1.5 to 5 years [7] to assess child behavioral challenges, Generalized Anxiety Disorder (GAD-7) [8] for caregiver anxiety, Patient Health Questionnaire (PHQ-8) [9] for caregiver depression, and Perceived Stress Scale (PSS-4) [10] for caregiver stress. Caregivers completed measurements at baseline and 3 months after intervention completion.

To estimate intervention effect sizes, we used Cohen *d* or (standardized mean difference), for paired samples. Cohen *d* value cutoffs of 0.2, 0.5, and 0.8 are considered as small, medium, and large effect sizes, respectively. *P* values were constructed from the Wilcoxon signed-rank test since the variables were not normally distributed, with significance set at *P*<.05.

This study was approved by the host institution’s Institutional Review Board (22‐37781) as minimal risk research. Signed informed consent was obtained from all participating caregivers. Data was stored on secure institutional servers and deidentified prior to analysis. Participants could receive up to $190 for completing all study activities.

## Results

A total of 28 caregiver/child dyads were recruited; of these, 79% (n=22) preferred Spanish and 14% (n=4) preferred English. Median child age was 4.5 years (IQR 1.66) and 50% (n=14) were male. Participants who completed both pre and post data collection were included in this analysis.

Three months post-intervention, caregivers reported large reductions in anxiety and perceived stress compared to baseline (Table 1).

**Table 1.** Pre-post changes in caregiver behavioral health measures.

Construct	Measures	Participants, n^a^ (N=28)	Baseline scores median, (IQR)	Post intervention scores, median (IQR)	Cohen *d* (SMD)^b^	*P* value
Caregiver anxiety	GAD-7^c^	16	5.5 (1.75-9.25)	1.0 (0-4.25)	0.86	.01
Caregiver depression	PHQ-8^d^	4	8.0 (6.5-9.25)	5.5 (3.75-7.25)	0.63	.42
Caregiver-perceived stress	PSS)- 4^e^	18	7.0 (6-8)	4.0 (3-6)	0.92	.02

a Number of participants who completed both pre and postdata collection for that measure.

b SMD: Standardized mean difference.

c GAD-7: Generalized Anxiety Disorder-7-item.

d PHQ-8: Patient Health Questionnaire-8-item.

e PSS-4: Perceived Stress Scale-4-item.

Notably, the decrease in caregiver anxiety (GAD-7: *d*=0.86, *P*=.01) and caregiver-perceived stress (PSS-4: *d*=0.92, *P=*.02) suggested large and statistically significant intervention effect sizes for parental anxiety and perceived stress among RC participants. The PHQ-8 responses were limited as the full instrument was administered only if the PHQ-2 score exceeded 4, making it difficult to draw conclusions.

Similarly, moderate reductions were seen in postintervention measures of multiple child behavior challenges, including attention problems, aggression, externalizing problems, stress, and overall problems. The decrease in attention problems (*d*=0.72, *P*=.05) approached significance. Selected child behavior domains showing moderate postintervention score reduction are listed in Table 2.

All other CBCL domains showed small effect sizes (*d*<0.5), including emotionally reactive (0.27), anxious depressed (0.12), somatic complaints (0.46), withdrawn (0.36), sleep problems (0.16), internalizing problems (0.32), anxiety problems (0.02), autism spectrum problems (0.48), and oppositional defiant problems (0.40).

**Table 2.** Pre-post changes in the Child Behavior Checklist (CBCL).

Child behavior domain	Number of participants, n^a^ (N=28)	Baseline scores, median (IQR)	Post-intervention scores, median (IQR)	Cohen *d* (SMD)^b^	*P* value
Attention problems	11	62 (57.5-67)	53 (51.5-62)	0.72	.05
Aggressive behaviors	11	64 (51.5-70)	56 (51-61)	0.53	.20
Externalizing problems	11	64 (53-70)	56 (48.5-60)	0.70	.11
Overall problems	11	68 (53.5-72)	58 (49.5-66.5)	0.50	.07
Stress	11	70 (53-72)	53 (52-62.5)	0.74	.09
Depression	11	63 (53-69.5)	56 (50-61.5)	0.52	.09
ADHD^c^	11	64 (59-71)	57 (52-65.5)	0.72	.08

a Number of participants who completed both pre and postdata collection for that measure.

b SMD: Standardized mean difference.

c ADHD: Attention deficit hyperactivity disorder

## Discussion

Three months post-intervention, caregivers reported significant reductions in anxiety and perceived stress and moderate reductions in their children’s attention problems, aggressive behaviors, externalizing problems, total problems, and stress and depressive problems. The decrease in caregivers’ anxiety and perceived stress was significant (*P*<.05), and the reduction in child attention problems approached significance (*P*=.05). These findings suggest that participation in this group resilience intervention may help improve caregiver stress and anxiety and child behavior.

Study limitations include the small sample size and lack of a control group, which make the findings preliminary. Given these promising results, a randomized controlled trial is needed to confirm these intervention effects.

Given the age of these children, when mental health diagnoses are rare, most children would not otherwise be receiving mental health services. This pilot study indicates that similar primary care-based, preventative group interventions may offer meaningful improvements in caregiver and child behavioral health in the context of childhood adversity.

## Abbreviations

ACE: adverse childhood experiences
CBCL: Child Behavior Checklist
GAD-7: Generalized Anxiety Disorder -7 item (screening tool for anxiety)
PHQ-8: Patient Health Questionnaire (PHQ)-8 item (screening tool for depression)
PSS: Perceived Stress Scale
RC: Resilience Clinic
